# Post-Transcriptional HIV-1 Latency: A Promising Target for Therapy?

**DOI:** 10.3390/v16050666

**Published:** 2024-04-24

**Authors:** Mie Kobayashi-Ishihara, Yasuko Tsunetsugu-Yokota

**Affiliations:** 1 Department of Molecular Biology, Keio University School of Medicine, Tokyo 160-8582, Japan; 2 Research Institute, The World New Prosperity (WNP), Tokyo 169-0075, Japan

**Keywords:** HIV-1, post-transcriptional latency, restriction factors, latency reversing agents

## Abstract

Human Immunodeficiency Virus type 1 (HIV-1) latency represents a significant hurdle in finding a cure for HIV-1 infections, despite tireless research efforts. This challenge is partly attributed to the intricate nature of HIV-1 latency, wherein various host and viral factors participate in multiple physiological processes. While substantial progress has been made in discovering therapeutic targets for HIV-1 transcription, targets for the post-transcriptional regulation of HIV-1 infections have received less attention. However, cumulative evidence now suggests the pivotal contribution of post-transcriptional regulation to the viral latency in both in vitro models and infected individuals. In this review, we explore recent insights on post-transcriptional latency in HIV-1 and discuss the potential of its therapeutic targets, illustrating some host factors that restrict HIV-1 at the post-transcriptional level.

## 1. Introduction

Recent advances in combination antiretroviral therapy (ART) have substantially controlled the replication of Human Immunodeficiency Virus type 1 (HIV-1). However, the virus remains latent even during this treatment, leading to variable issues such as side effects, economic constraints, patient compliance, and the risks associated with HIV-associated neurocognitive disorders (HAND) [1]. Consequently, there is a high demand for new approaches capable of achieving functional or complete cures for HIV-1 infections.

One such approach to eliminate latent viruses is the shock-and-kill strategy [2]. This method involves using drugs known as latency reversing drugs (LRAs) to reactivate the dormant viruses, and then the infected cells can be eliminated using ART and antiviral immune responses. Another strategy known as the “block-and-lock” strategy was later proposed [3]. This approach aims to induce a state of deep latency by using drugs called latency-promoting agents (LPAs) [4,5,6]. Recently, a novel concept called “rinse-and-replace” was proposed by Grossman and colleagues [7]. According to this theory, polyclonal activation of HIV-infected memory CD4+ T cells by LRAs leads to their physiological death. Subsequently, homeostatic controls replenish this T cell pool with newly developed uninfected memory CD4+ T cells.

Despite significant research efforts to develop practical approaches for HIV-1 latency, particularly focusing on LRAs that can reactivate HIV-1 transcription, the current LRAs have shown limited success in achieving complete latency reversal in people with HIV (PWH) [1,8,9,10,11]. This limitation is attributed to the heterogeneous nature of individual reservoirs, where various viral processes besides transcription may remain silent [1,12]. Therefore, it is crucial to understand HIV-1 latency beyond the transcriptional level for the development of an effective cure. This review will primarily focus on the post-transcriptional (PT) latency of HIV-1 and discuss several restriction factors that could be potential therapeutic targets to overcome this form of latency.

## 2. Post-Transcriptional HIV-1 Latency: A Robust Latency Style in Infected Individuals?

**Figure 1:**
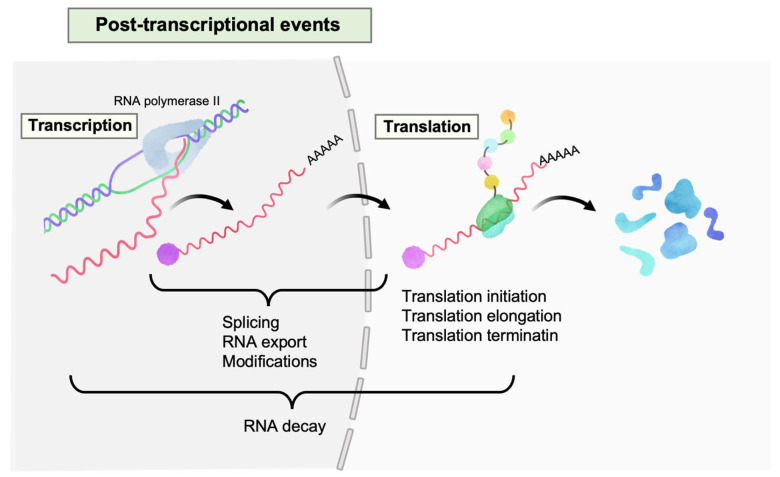
Post-transcriptional events in HIV-1 gene expression. Multiple events occur on HIV-1 RNAs (colored in pastel red) after transcription, such as splicing, nucleocytoplasmic export, modifications, translation (initiation, elongation, and termination), and RNA decay.

HIV-1 exploits multiple host post-transcriptional (PT) regulatory systems as depicted in Figure 1. The interplay between these processes and HIV-1 replication is well reviewed elsewhere [13,14,15,16]. In brief, processes such as splicing, modifications, and nucleocytoplasmic export increases the functional capacity of HIV-1, while RNA decay and translation systems are necessary for efficient virus production. Some regulation machinery like RNA decay are co-opted by HIV-1 for its optimized replication, despite their fundamental antiviral functions [13]. Thus, the loss or inhibition of these regulatory systems can lower HIV-1 protein expression, resulting in the failure of virion production despite active viral transcription.

The concept of PT latency represents the infection status described above. This is also recognized as a part of persistent infections (reviewed in [17]). However, the majority of persistent HIV-1 hardly produces virions compared to the other persistent viruses that show chronic infections. This was first evidenced in PWH by Fisher et al. who succeeded in measuring cellular HIV-1 RNA in the peripheral blood mononuclear cells (PBMC) of PWH on suppressive ART, despite the RNA from the cell-associated viral particles being barely detectable [18,19]. Similar findings have been shown in resting CD4+ T cells from infected individuals, a major subset of latently infected cells [20,21], which collectively suggest the presence of cells with post-transcriptionally latent HIV-1.

Studying HIV-1 latency is challenging, particularly due to technical barriers related to the scarcity of latently infected cells [20]. Despite these challenges, various studies have suggested a prevalence of PT HIV-1 latency in in vitro latency models [22,23] and in PWH [9,18,19,20,21,24,25,26]. The studies generally demonstrated poor latency reversal even under the presence of transcriptional activation. For instance, we previously established a latency model with primary CD4+ T cells cultured for a prolonged period [23]. This model showed mere changes in intracellular viral protein levels by the treatment of LRAs despite a significant increase in RNA levels. Prominently, Maria’s group developed the FISH-flow assay, which is capable of detecting HIV-1 RNA and Gag-p24 protein at a single-cell level [27]. With this approach, they characterized reactivity against several LRAs in patient PBMCs with ex vivo treatments [9]. The results revealed that less than 10% of latently infected cells expressed the p24 protein, even with drugs that significantly induced HIV-1 RNA expression. Furthermore, some LRAs exhibited weak correlations between the frequency of cells expressing HIV-1 RNA and p24, suggesting incomplete reactivation of post-transcriptionally latent HIV-1 by these treatments.

Yukl et al. conducted a study that compared different viral RNA species using droplet digital PCR. They identified a strong blockade during transcriptional elongation and the PT steps (polyadenylation and splicing) in CD4+ T cells from PWH on ART [24]. Despite being on suppressive ART, between 89 and 100% of the infected CD4+ T cells were estimated to have HIV-1 transcripts, a finding consistent across several studies [19,24]. However, these studies remain to be questioned regarding whether this refractory to latency is due to the viral defects, as most HIV-1 proviruses in ART-treated individuals are estimated to have critical mutations [12,28,29]. Furthermore, even during ART, transcripts may originate from the cells reactivated from latency, as well as newly infected cells, as minor viral replication may occur in tissues where antiretroviral drugs do not reach a sufficient level [30,31,32,33]. Therefore, precise techniques are required to distinguish the origins of transcripts in this scarce condition and clarify the extent to which PT latency affects PWH. Nevertheless, these observations collectively support the notion that PT latency affects individuals undergoing treatment, suggesting that targeting PT latency could contribute to an effective cure.

## 3. Mechanisms of Post-Transcriptional Latency in HIV-1

Controlling PT latency not only holds promise for HIV-1 eradication or containment but also offers the potential to improve the immune condition. The persistence of viral transcripts results in chronic immune activation, exacerbating pathogenesis and mortality among infected individuals [34,35,36]. Therefore, achieving effective targeting of PT latency in HIV-1 requires a comprehensive understanding of its underlying mechanisms.

As previously discussed, PT latency can arise from two primary mechanisms, namely the loss or inhibition of host PT regulatory systems. This notion is supported by compelling evidence from reports showing the absence of essential PT regulators in resting CD4+ T cells. For instance, a transcriptome-wide study identified the significant downregulation of genes related to RNA processing and modifications in resting CD4+ T cells from PWH [21]. Additionally, the scarcity of several RNA exporters, such as PTB or MATR3, causes nucleus retention of HIV-1 RNAs in resting CD4+ T cells [25,37]. These expression levels were correlated with the poor efficiency of LRAs, indicating the pivotal role of host PT regulatory systems in HIV-1 latency. Thus, these findings provide valuable insights into potential targets for effective intervention.

**Figure 2:**
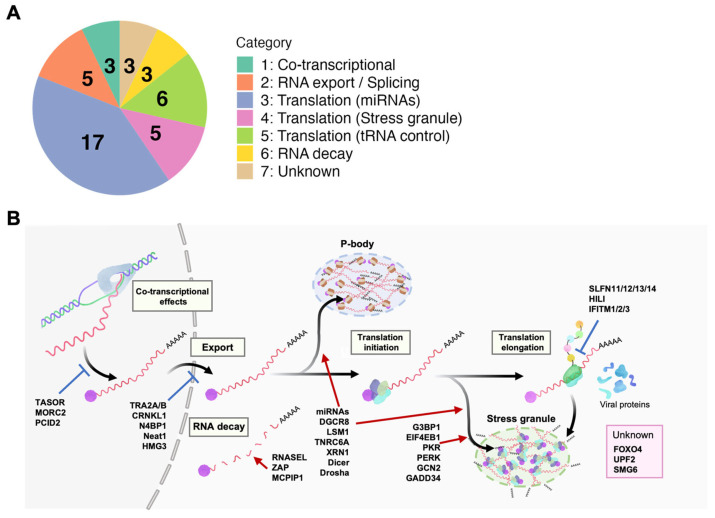
Host factors downregulating HIV-1 replication at the post-transcriptional level. (**A**) Functional categories and their numbers of identified (potential) restriction factors. MicroRNAs (miRNAs) that directly inhibit HIV-1 expression are included as restriction factors here. The plot was created by ggplot2 (3.4.3) R-package. (**B**) Graphical summary of the restriction factors. The numbers correspond to the categories shown in A. Detailed information is provided in Table 1.

Furthermore, a subset of restriction factors has been reported to inhibit HIV-1 by acting on PT regulation (reviewed in [16]). Those PT restriction factors are activated by host innate immunity, representing a frontline of the host’s antiviral defense mechanism. A comprehensive review of the literature has identified a diverse array of PT restriction factors, now encompassing over 40 known factors (Table 1) [38,39,40,41,42,43,44,45,46,47,48,49,50,51,52,53,54,55,56,57,58,59,60,61,62,63,64,65,66,67,68,69,70,71,72,73,74,75,76,77,78,79,80,81,82,83,84]. Categorization of these factors into distinct groups based on their inhibitory mechanisms offers a structured framework for understanding their roles in HIV-1 latency (Figure 2). Notably, a significant number of these factors targets HIV-1 translation (categories 3–5), emphasizing the importance of this process in PT latency.

Taken together, the modification of the dynamics between these two mechanisms may have an impact on the control of PT latency in HIV-1.

## 4. Host Factors That Restrict HIV-1 at the Translational Level

Translation regulation involves diverse factors, including the formation of granules such as processing (P)-bodies and stress granules (SGs), which can limit mRNA translation by spatial containments via phase separation [85]. MicroRNAs (miRNAs) play a crucial role in this process by transporting the targeted mRNAs to these granules (reviewed in [86]). Additionally, several restriction factors can inhibit the function of ribosomes or tRNAs, illustrated by those controlling tRNA abundance.

In this section, we will discuss miRNAs/P-bodies (category 3), SGs (category 4), and tRNA controllers (category 5) in HIV-1 PT latency, along with their potential as targets for therapeutic developments.

### 4.1. MicroRNAs

MicroRNA (miRNA)-mediated RNA silencing is a crucial mechanism for regulating gene expression at the PT level [87]. The process of miRNA biogenesis involves two RNase III enzyme complexes, Drosha/DGCR8 and Dicer/TRBP/PACT, localizing in the nucleus and cytoplasm, respectively. These proteins generate pre-miRNAs from non-coding RNAs transcribed by RNA polymerase II. The pre-miRNAs are further incorporated into the RNA-induced silencing complex (RISC), which is composed of Dicer/TRBP/PACT plus Argonaute proteins. Thereafter, the pre-miRNAs are processed into mature miRNAs. This RISC–miRNA complex is delivered to the processing (P)-bodies or SGs (see also Section 4.2) and recruit the target mRNA, leading to its degradation or translation repression. Thus, miRNAs are key mediators of PT regulations in gene expression [86].

According to a study by Chable-Bessia et al., HIV-1 RNAs co-localized with RISC components. The knockdown of DGCR8 and P-body component RCK/p54 (DDX6) resulted in virus reactivation in PBMCs of PWH on suppressive ART [79]. This strongly indicates that miRNAs play a role in HIV-1 latency establishment through the direct targeting of HIV-1 mRNAs. The report by Huang et al. identified a cluster of miRNAs, including miR-28, miR-125b, miR-150, miR-223, and miR-382, which is enriched in resting primary CD4+ T cells and that target HIV-1 mRNA [71]. Furthermore, specific inhibitors against these miRNAs were found to reactivate virus production in resting primary CD4+ T cells either transfected with infectious HIV-1 clones or from PWH on suppressive ART. These miRNAs are found to have similar roles in monocytes and macrophages [42]. However, a recent observation has shown that this series of miRNAs have little contribution to HIV-1 latency [88]. Another study identified miR-196 and miR-1290, which target and regulate HIV-1 RNA by profiling miRNA expression in latent or active HIV-1 infections [43]. This finding has not been followed by further reports. Therefore, it remains unclear as to whether and how these miRNAs work for the maintenance of HIV-1 latency.

miR-29a has been a well-studied miRNA directly targeting HIV-1. This cellular miRNA was initially predicted as a potential miRNA targeting HIV-1 and was validated to enhance the association of viral RNA with RISC proteins, leading to its degradation [56]. Later, Patel et al. observed increased miR-29a levels during latency and a decrease following active HIV-1 replication, indicating its association with viral latency [52]. Additionally, IL-21 treatment was found to upregulate the expression of miR-29a clusters, which effectively limited early HIV-1 infection in vitro [83]. Therefore, gaining a comprehensive understanding of the regulatory role of miR-29a in HIV-1 infection may provide valuable insights for the development of an effective cure.

miRNAs can suppress HIV-1 replication indirectly by targeting the cellular mRNAs essential for HIV-1 replication, such as viral entry and transcriptional activation (reviewed in [89]). For instance, the expression profile study of miRNAs identified 26 dysregulated miRNAs in an in vitro latency model with primary CD4+ T cells [90]. Coupled with transcriptome and pathway analyses, this study revealed the enrichment of target mRNAs associated with the p53 signaling pathway, which has previously been shown to reactivate latent HIV-1 [91]. In monocytes and monocyte-derived dendritic cells, HIV-1 expression is naturally suppressed by a lack of the HIV-1 transcription activator, Pur-alpha, which is regulated by a subset of miRNAs, including miR-15a and miR-15b [92]. Recently, one report showed that miR-642a-3p suppresses HIV-1 transcription through the degradation of mRNA encoding for AFF4, a protein essential for transcriptional elongation. Notably, HIV-1 Gag has been shown to sequester miR-642-3p, thereby thwarting its suppressive regulation by forming a complex with Dicer [82].

In addition to the suppressive role in HIV-1 replication, some miRNAs can instead promote HIV-1 replication, counteracting the innate immune activation. TGF-β is one of cytokines involved in the innate immune response and suppresses HIV-1 replication [93,94], while miR-155 indirectly reactivates HIV-1 latency through the inhibition of TGF-β production from the cervical epithelial cells [95]. Furthermore, miRNA-containing extracellular vesicles (EVs) [96,97] have been proposed to reactivate the virus in latently infected cells via miRNA transfer [98]. Mechanistically, plasma EVs from HIV-1-infected individuals contain abundant miR-139-5p, which can reduce the expression of FOXO1 upon transfer to treated cells [98]. FOXO1 is known to promote HIV-1 latency by suppressing ER stress in T cells [99], thus the decrease in FOXO1 may reverse HIV-1 latency by the EVs of PWH. In summary, these regulatory pathways appear to be involved in the pathogenesis of HIV-1 [100], although their relevance remains elusive and is thus an essential question.

In conclusion, miRNAs exhibit complex effects, serving to both restrict and indirectly promote HIV-1 replication. miRNAs directly targeting HIV-1 represent potentially specific and feasible targets for therapeutic interventions. However, it is important to note that since a single miRNA typically targets multiple mRNA species, the potential for unintended side effects must be carefully considered [101]. Further studies are needed to fully understand the role of miRNAs in combating HIV-1 latency and to establish a therapeutic foundation.

### 4.2. Stress Granules

SGs are condensed, phase-separated cytoplasmic granules that are formed in stress responses to sequestrate non-translating mRNA molecules [85]. Their formation is initiated by various factors, such as PKR (EIF2AK2), PERK (EIF2AK3), and GCN2 (EIF2AK4). These factors recognize and are activated by viral double stranded RNAs (dsRNAs), as well as phosphorylate translation initiators, eIF2α (reviewed in [102]). The inactivated eIF2α is then unable to carry the Met tRNA on the ribosome, leading to the blockage of global translation initiation. As a result, non-translated mRNAs are gathered with the SG proteins, including G3BP1 and TIAR, leading to the formation of SGs.

In the HIV-1 infection, PKR interacts with the dsRNA structure in HIV-1 RNA called the transactivation-responsive region, TAR. This interaction blocks the translation initiation process by inactivating eIF2α [55,57,72,84]. Additionally, GCN2 suppresses HIV-1 translation by hindering eIF2α activity [75]. PERK can also inactivate eIF2α upon HIV-1 infection, but it is unlikely to affect HIV-1 translation levels [99,103,104,105]. Knocking down the SG protein G3BP1 reactivates HIV-1 in primary CD4+ T cells and macrophages [77], suggesting that SG control may also alter PT latent infections. However, this series of antiviral mechanisms can be counteracted by HIV-1. For example, HIV-1 Tat can mask the TAR from PKR recognition [53] and also impede its activity through the use of PACT, ADAR1, and TRBP [48,55,106,107,108]. HIV-1 Gag and the nucleocapsid protein (NC) can inhibit SG assembly by binding the host factors eEF2 and Stau1, respectively [44,109,110]. Thus, these viral counteractions create the complex interplay among HIV-1 infection, host factors, and SG formation.

Within the intricate landscape of these studies, recent findings have illuminated the role of SG assembly in HIV-1 replication. It has been revealed that HIV-1 RNAs rely on zinc (Zn^2+^) chelation, altering the phase separation property of Gag and NC [58]. Both Gag and NC possess two zinc-finger (ZnF) domains, and without Zn^2^ chelation, Gag can phase-separate viral RNAs from SGs, thereby facilitating viral propagation. Furthermore, NC inhibits G3BP1 to block the sequestration of HIV-1 RNA in SGs, with NC mutants at the ZnF domain losing this inhibitory effect. Remarkably, Zn^2+^ chelation has been found to decrease HIV-1 production. Consequently, targeting the NC–ZnF domains by specific compounds may enhance the SG assembly of HIV-1 RNAs, offering potential efficacy for a functional cure [58].

In addition to the cascade that leads to the SG formation, the stress response pathway triggers multiple networks, including the endoplasmic reticulum (ER) stress response [102,111,112]. When the protein PERK senses ER stress and allows for the phosphorylation of eIF2α, a transcription factor called ATF4 is selectively translated and triggers the signaling of cell death. Additionally, ATF4 can stimulate the transcription of HIV-1 [113,114,115]. Ex vivo treatment with HA15, which is a selective inducer of the ER stress response, results in HIV-1 reactivation and a significant decrease in reservoir size [116]. These findings raise important questions regarding the impact of selectively activating or inhibiting different parts of the stress response on HIV-1. One such question is whether the targeting of the stress response pathway leads to more effective PT containment or reactivation of latent HIV-1. It is also important to understand how HIV-1 exploits these intricate pathways. A comprehensive understanding of SG formation and stress response in HIV-1 latency may lead to new therapeutic applications.

### 4.3. tRNA Controllers

tRNAs are critical players in translation, with the decoding of RNA codons to amino acids. Therefore, the changes in tRNA abundance affect translation levels. For instance, carcinogenesis [117,118,119] and virus infections (reviewed in [120]) can influence cellular tRNA abundance, which consequently slows down codon-biased translation. Notably, HIV-1 transcripts have AU-rich sequences and show frequent A-ending codons, which are less common in humans [121]. Category 5 restriction factors can impede viral translation in a codon-usage-dependent manner by modifying the host’s tRNA pool (Figure 2 and Table 1).

The SLFN family proteins, comprising seven members, have been mostly studied in this regard. Among them, SLFN11, 12, and 14 have been identified to show codon-usage-dependent restrictions [45,60,65,68]. SLFN11 and 12 cleave tRNAs against the leucine (UUA) codon, which leads to ribosomal stalling on the HIV-1 transcripts [64,68,122,123]. Knocking down SLFN11 or 12 in HIV-1 latently infected T cells showed elevated levels of latency reversal, indicating the contribution to HIV-1 latency [68]. SLFN14 likely impairs HIV-1 expression by degrading ribosomes engaged in codon-biased transcripts [45]. Conversely, SLFN13 cleaves various tRNAs and rRNAs, thereby inhibiting global translation in infected cells beyond the influence of codon biases [39]. Meanwhile, SLFN5 suppresses HIV-1 transcription, as it functions distinctly compared to the other SLFNs [124]. Understanding the interplay of these SLFNs may hold hints for novel interventions.

Additionally, HILI, a human PIWI-like protein [125], and the IFITM family proteins inhibit HIV-1 translation in a codon-usage-dependent manner [51,66]. While HILI can sequester tRNAs against arginine (AGA) and isoleucine (AUA) codons, IFITM proteins, known for blocking HIV-1 entry [126], also hinder its translation, hinting at a role in tRNA control. However, it is still unclear how much these factors contribute to the latency of HIV-1 infection.

Overall, tRNA control can play a critical role in the establishment and maintenance of PT latency in HIV-1. Given their capacity for biased translation control, these factors could be promising therapeutic targets with high specificity against latent HIV-1. However, the relationship among these factors and their precise impact on PT latency remain to be fully elucidated, underscoring the need for continued research in this area.

## 5. Conclusions and Future Perspectives

**Figure 3:**
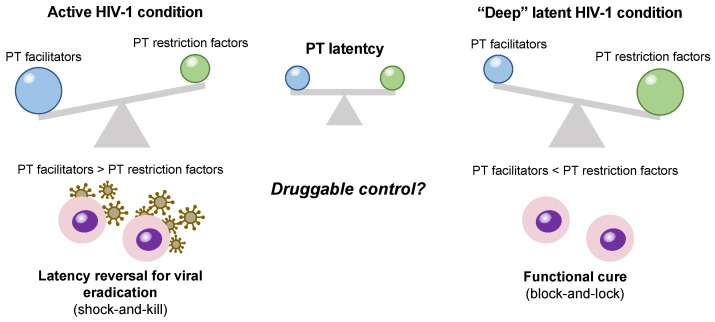
Balance between facilitator and restriction factors in post-transcriptional HIV-1 latency. PT facilitators (e.g., PTB and MATR3) promote HIV-1 expression at the post-transcriptional level, while PT restriction factors (e.g., SLFN12) inhibit this process. Modulating the functional equilibrium between these factors using drugs could enhance proposed therapeutic strategies such as shock-and-kill or block-and-lock.

A review of past reports indicates considerable evidence that PT regulations have a major influence on establishing and maintaining HIV-1 latency. This PT latency can be achieved by the lack of essential factors and the activation of restriction factors. Therefore, controlling the balance between these two mechanisms could lead to a better treatment outcome (Figure 3). However, its molecular mechanisms are very complex, as HIV-1 has evolved to overcome host restrictions; it is sometimes difficult to conclude whether the PT regulator is a “facilitator” or a “restriction factor”, represented as the SG factors (see Section 4.2).

Considering this complexity, comprehensive studies of these factors are necessary for effective intervention, including the elucidation of viral counteractors. Encouragingly, several druggable PT inhibitors have already been reported to be effective in suppressing HIV-1 in vitro or ex vivo [127,128,129,130,131,132]. Thus, the pursuit of PT latency control emerges as a pivotal strategy in combating HIV-1 latency, warranting further careful investigation on its intricacies.

## Figures and Tables

**Table 1:** List of (potential) restriction factors.

Category	Restriction Factor	Mechanisms	Reference(s)	Counteractor(s)
1	TASOR	Recruits H3K9me3 in HIV-1 proviral region and brings RNase CNOT1 and exosome PAXT complex onto nascent HIV-1 RNAs	[59,67]	Vpr
1	MORC2	Recruits HIV-1 LTR and brings RNase CNOT1 and PAXT complex onto the nascent HIV-1 RNAs	[67]	- ^1^
1	PCID2	Interacts with TREX2 complex, which is necessary for transcription initiation and alternative RNA splicing. The complex binds HIV-1 LTR to repress HIV-1 expression both at the transcriptional and post-transcriptional levels	[76]	- ^1^
2	TRA2A/B	Reduces HIV-1 Gag/Env expression by affecting splice site usage and RNA export. Conversely reported to augment HIV-1 replication via facilitation of vpr mRNA processing	[50,74]	- ^1^
2	CRNKL1	Enhances nuclear retention of unspliced HIV-1 RNAs	[41]	- ^1^
2	N4BP1	Exhibits RNase activity against HIV-1 RNAs, localizing in nucleolus (PML bodies)	[40]	- ^1^
2	Neat1	Inhibits nucleocytoplasmic export of unspliced HIV-1 RNA via paraspeckle formation	[38,63,80]	Rev
2	HMGB3	Inhibits Tat mRNA processing and translation by binding TIM–TAM region of HIV-1 RNA	[69]	- ^1^
3	RCK/p54 (DDX6)	P-body components. Downregulates HIV-1 gene expression by preventing viral mRNA association with polysomes	[79]	- ^1^
3	LSM1			
3	XRN1			
3	TNRC6A (GW182)	RISC component. Downregulates HIV-1 gene expression by preventing viral mRNA association with polysomes	[79]	- ^1^
3	Dicer	RISC component and miRNA processing enzyme (RNase III). Inhibits HIV-1 gene expression and essential host factors for HIV-1 replication	[56,82]	Gag
3	DGCR8	miRNA processing factors. Inhibits HIV-1 gene expression by preventing viral mRNA association with polysomes	[79]	- ^1^
3	Drosha		[56,79]	- ^1^
3	MIR125B2	MicroRNAs that inhibit virus production by directly binding HIV-1 RNAs	[42,71]	- ^1^
3	MIR150			
3	MIR28			
3	MIR223			
3	MIR382			
3	MIR29A		[52,56,83]	
3	MIR29B1		[56]	
3	MIR29C			
3	MIR 196b		[43]	
3	MIR 1290			
4	G3BP1	Sequesters viral RNAs by SG formation	[44,58,77]	Gag (Pr55, NC)
4	EIF4EBP1 (4EBP1)	Its hypophosphorylated form binds eIF4E−5′ cap of HIV-1 RNA complex, inhibiting the translation of HIV-1 RNA by SG assembly	[78]	Gag
4	PKR (EIF2AK2)	Senses viral RNAs and enhances integrated stress response (ISR) for global translational shutdown and SG formation	[48,53,55,57,72,84]	Tat, Gag
4	GCN2 (EIF2AK4)	Senses viral RNAs and enhances integrated stress response (ISR) for global translational shutdown and SG formation	[75]	Pol (Pro)
4	GADD34	Interacts with HIV-1 TAR RNA and inhibits its translation independently of eIF2a inactivation	[70]	- ^1^
5	SLFN11	Cleaves type II tRNA degradation, such as leucine (UUA) tRNA, leading to inhibition at the translation elongation of HIV-1	[60,64,65,68]	- ^1^
5	SLFN12	Cleaves leucine (UUA) tRNA, leading to inhibition at the translation elongation of HIV-1	[68]	- ^1^
5	SLFN13	Cleaves rRNAs and tRNAs, driving global translation inhibition including HIV-1	[39]	- ^1^
5	SLFN14	Impedes HIV-1 protein expression by degrading ribosomes engaged in codon-biased transcripts	[45]	- ^1^
5	IFITM1/2/3	Inhibits HIV protein synthesis in a codon-usage manner	[66]	Nef
5	HILI	Inhibits HIV-1 translation by sequestering arginine (AGA) and isoleucine-AUA tRNAs	[51]	- ^1^
6	RNASEL	Recognizes 2′-5′-linked oligoadenylated HIV-1 RNA and degrades it	[49,61]	- ^1^
6	ZAP	Degrades cytoplasmic HIV-1 RNAs. Interacts with CG dinucleotide containing RNAs	[46,73,81]	- ^1^
6	MCPIP1 (ZC3H12A)	Decreases steady state level of HIV-1 RNAs by its endoribonuclease activity	[62]	- ^1^
7	FOXO4	Destabilizes Tat mRNA, repressing Tat protein levels. This consequently decreases transcription levels of HIV-1	[54]	- ^1^
7	UPF2	Inhibits HIV-1 reactivation post transcription. This needs interaction with UPF1	[47]	- ^1^
7	SMG6			

^1^ “-” represents counteractor(s) that have yet to be determined.

## Abbreviations

HDAC	Histone deacetylase
PKC	Protein kinase C
PTB	Polypyrimidine tract-binding protein
MATR3	Matrin3
Drosha	Drosha ribonuclease III
DGCR8	DiGeorge syndrome critical region 8
Dicer	Dicer ribonuclease III
TRBP	TAR-RNA binding protein
PACT	Protein kinase R activating protein
DDX6	Dead-box helicase 6
Pur-alpha	Purine-rich element binding protein alpha
AFF4	ALF transcription elongation factor 4
TGF-β	Transforming growth factor-β
FOXO1	Forkhead box O1
PKR	Protein kinase R
PERK	PKR-like ER kinase
GCN2	General control nonderepressible 2
eIF2α	Eukaryotic translation initiation factor 2α
G3BP1	Ras GTPase-activating protein-binding protein 1
TIAR	T-cell intracellular antigen 1 (TIA1)-related protein
ADAR1	Adenosine deaminase acting on RNA 1
eEF2	Eukaryotic elongation factor 2
Stau1	Staufen 1
ATF4	Activating transcription factor 4
SLFN	Schlafen
